# NMR Solution Structure of a Chymotrypsin Inhibitor from the Taiwan Cobra *Naja naja atra*

**DOI:** 10.3390/molecules18088906

**Published:** 2013-07-26

**Authors:** Yi-Jan Lin, Teppei Ikeya, Peter Güntert, Long-Sen Chang

**Affiliations:** 1 Graduate Institute of Natural Products and Center of Excellence for Environmental Medicine, Kaohsiung Medical University, No.100, Shi-Chuan 1st Road, San-Ming District, Kaohsiung 807, Taiwan; 2 Department of Chemistry, Graduate School of Science and Engineering, Tokyo Metropolitan University, 1-1 Minami-ohsawa, Hachioji, Tokyo 192-0397, Japan; E-Mail: tikeya@tmu.ac.jp; 3 Institute of Biophysical Chemistry, Center for Biomolecular Magnetic Resonance, Goethe University Frankfurt am Main, Max-von-Laue-Str. 9, 60438 Frankfurt am Main, Germany; E-Mail: guentert@em.uni-frankfurt.de; 4 Frankfurt Institute for Advanced Studies, Goethe University Frankfurt am Main, Ruth-Moufang-Str. 1, 60438 Frankfurt am Main, Germany; 5 Institute of Biomedical Sciences, National Sun Yat-Sen University, Kaohsiung 804, Taiwan; E-Mail: lschang@mail.nsysu.edu.tw

**Keywords:** *Naja naja atra*, snake venom, chymotrypsin inhibitor, NACI, BPTI, NMR spectroscopy, NMR structure determination, disulfide bond isomerization

## Abstract

The Taiwan cobra (*Naja naja atra*) chymotrypsin inhibitor (NACI) consists of 57 amino acids and is related to other Kunitz-type inhibitors such as bovine pancreatic trypsin inhibitor (BPTI) and *Bungarus fasciatus* fraction IX (BF9), another chymotrypsin inhibitor. Here we present the solution structure of NACI. We determined the NMR structure of NACI with a root-mean-square deviation of 0.37 Å for the backbone atoms and 0.73 Å for the heavy atoms on the basis of 1,075 upper distance limits derived from NOE peaks measured in its NOESY spectra. To investigate the structural characteristics of NACI, we compared the three-dimensional structure of NACI with BPTI and BF9. The structure of the NACI protein comprises one 3_10_-helix, one α-helix and one double-stranded antiparallel β-sheet, which is comparable with the secondary structures in BPTI and BF9. The RMSD value between the mean structures is 1.09 Å between NACI and BPTI and 1.27 Å between NACI and BF9. In addition to similar secondary and tertiary structure, NACI might possess similar types of protein conformational fluctuations as reported in BPTI, such as Cys14–Cys38 disulfide bond isomerization, based on line broadening of resonances from residues which are mainly confined to a region around the Cys14–Cys38 disulfide bond.

## 1. Introduction

The *Naja naja atra* chymotrypsin inhibitor (NACI) is a protease inhibitor present in the venom of the Taiwan cobra(*Naja naja atra*). This chymotrypsin inhibitor belongs to the class of non-neurotoxic snake Kunitz/BPTI inhibitors [1], which are different from their snake Kunitz/BPTI neurotoxic homologues, such as dendrotoxins, calcicludine and the B chain of β-bungarotoxin which act as Ca^2+^ or K^+^ channel blockers [2]. A comparative analysis of the Kunitz/BPTI inhibitors and their neurotoxic homologues indicated that residue changes in the active sites result in both conformational adjustment and functional divergence [3,4,5]. The Kunitz domain recognizes one or more proteases through a set of 10–14 residues that are located mainly in the primary binding loop and partly in the second loop [6]. P1, the reactive residue, is located in the primary binding loop, which is important for the binding or recognition between the Kunitz domains and proteases. If P1 is Lys or Arg, the protein tends to inhibit trypsin, whereas if P1 is Leu, Phe, Tyr, Met, or Trp, the protein tends to inhibit chymotrypsin [7]. The second loop also contributes to specificity [6]. Structurally, the second loop is connected to the primary binding loop via a disulfide bridge, and together, these loops form an active site that is thought to be important for the interaction with the protease. In addition to the P1 residue, residues surrounding the active site are important as well. Variable residues located in the weak contact loop provide different interactions with various proteases. Hence, these surrounding residues are important for inhibitor protease specificity as well [3,7].

NACI consists of 57 amino acids, six of which are cysteines. The locations of the six cysteine residues are comparable to those of the cysteines in the homologous snake Kunitz/BPTI protein family [3]. Because P1 of NACI is Phe, NACI is classified as a chymotrypsin inhibitor. In addition to the P1 residue, based on the amino acid sequence alignment of NACI with other Kunitz/BPTI protease inhibitors, some conserved residues, such as Gly12, Tyr23, Phe33, Tyr35, Gly37, Asn43, and Phe45 probably play important roles in contact with the protease [3,8].

The three-dimensional structure of one chymotrypsin inhibitor, BF9, from the Elapid snake *Bungarus fasciatus* has been reported [5]. BF9 contains 65 amino acids with three disulfide bonds and was shown to consist of a double-stranded antiparallel β-sheet and one α-helix. Cys14 of BPTI is adjacent to the P1 position, Lys15, and forms a disulfide bond with Cys38, while the corresponding P1 position in BF9 is Asn17 and the corresponding disulfide bond is Cys16–Cys40 based on sequence alignment of BPTI and BF9 [5]. Due to isomerization of the Cys14–Cys38 disulfide bond, two conformational isomers with different chirality were observed in BPTI in the NMR spectra [9,10]. The population of the two isomers is temperature-dependent. Conformational changes by the Cys14–Cys38 disulfide bond isomerization cause NMR line broadening. It was reported that some ^1^H resonances of residues 14–18 in BPTI are broadened at 36 °C and only few NOEs could be assigned [9]. In addition, increased transverse ^13^C relaxation rates were observed for the C^α^ resonances of Cys14 and Cys38 [9]. Furthermore, it was reported that isomerization of the Cys14 side chain between χ^1^ rotamers is faster than the corresponding Cys38 isomerization [10]. 

## 2. Results and Discussion

### 2.1. Chemical Shift Assignment

**Figure 1:**
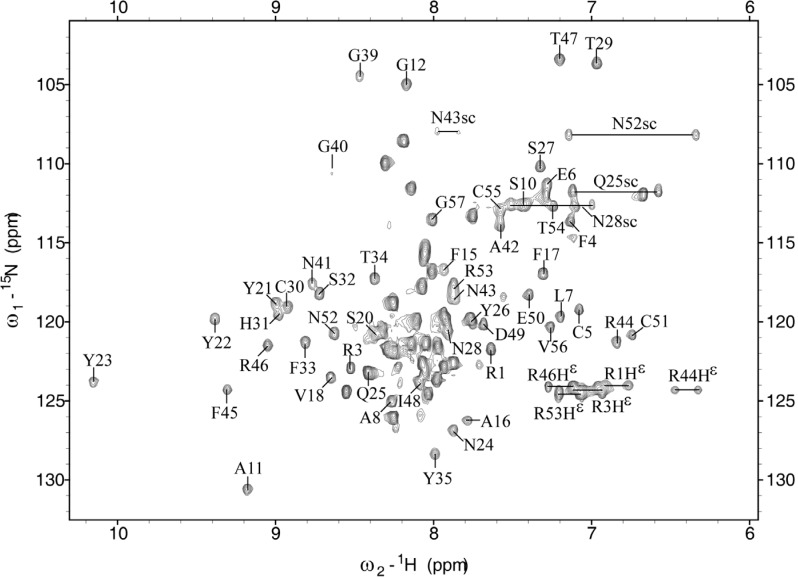
Two-dimensional [^15^N,^1^H]-HSQC spectrum of NACI. Cross peaks are labeled with the one letter code and the sequence number of the corresponding amino acid. The regions of ε-proton resonances of arginine residues were not decoupled in order to distinguish these resonances from other amide proton resonances.

The protein construct used for the structure determination comprised 89 amino acid residues, including 32 non-native residues at its N-terminus that are related to the expression system. The C-terminal 57 residues constitute the NACI protein, for which 94.8% of the backbone amide protons and the non-labile protons were assigned, except residues Ser13 and Ser36–Cys38 as well as H^N^ of Cys14, H^ε1^ of His31, and the aromatic protons of Phe45. The overall completeness of resonance assignments is well above the threshold required for automated NOESY cross peak assignment [11,12]. Among the labile side-chain protons, the ε-proton resonances of all arginine residues and the amide groups of asparagine and glutamine residues, except Asn24 and Asn41, were assigned. Several unusual chemical shifts were observed in NACI. For example, H^β^ protons (0.24 and 0.34 ppm) of Pro9 and H^α^ protons (2.90 and 1.65 ppm) of Ile48 and Cys51 were unusually shifted to upfield regions, which was also found in the corresponding residues of BF9 [5]. Based on the three-dimensional structure of NACI, the observed unusual chemical shifts can be attributed to aromatic ring current effects. Figure 1 shows a well-dispersed and assigned [^1^H,^15^N]-HSQC spectrum of NACI.

The unassigned signals in the HSQC spectrum correspond to the 32 non-native residues that are derived from the expression plasmid. Regions from labile side-chain protons of arginine residues were not decoupled for the fast identification of the ε-proton resonances. The unassigned signals of the backbone amide protons from non-native residues are distributed from about 7.5 to 8.5 ppm, suggesting that the non-native residues do not form any well-defined structure. Signals from residues around the Cys14–Cys38 disulfide bond are missing, including residues Ser13, Ser36–Cys38, and H^N^ of Cys14. The intensity of the ^13^C^β^ signal of Cys14 is weak, whereas the ^13^C^β^ resonances of the other four cysteine residues Cys5, Cys30, Cys51, and Cys55 showed intense signals, which is in agreement with the report that increased transverse ^13^C relaxation rates were observed for α-carbon resonances of residues Cys14 and Cys38 in BPTI [9]. Except for the unassigned ^13^C^β^ resonance of Cys38, the other five ^13^C^β^ chemical shifts of Cys5, Cys14, Cys30, Cys51, and Cys55 range from ~39 to ~50 ppm, which is consistent with the presence of three disulfide bonds in NACI.

### 2.2. Structure Determination and Solution Structure of NaCl

Excluding the unassigned residues, Ser13 and Ser36–Cys38, about 20 NOE distances restraints per residue, including 377 long-range distance restraints between protons five or more residues apart in the sequence, were used in the final structure calculation with CYANA [13,14]. 

The three-dimensional structure of NACI was examined using PROCHECK-NMR [15] and MOLMOL [16]. The 20 energy-minimized conformers are well-defined with RMSD values to the mean coordinates of 0.37 Å for the backbone and 0.73 Å for all heavy atoms in the region of residues 1–57. Table 1 lists structural statistics of the final 20 energy-minimized conformers of NACI.

**Table 1:** Structural statistics for the NMR solution structure of NACI.

NOE distance restraints:	
Number	1075
Intraresidual, |*i* – *j*| = 0	121
Sequential, |*i* – *j*| = 1	324
Medium range, 1 < |*i* – *j*| < 5	253
Long range, |*i* – *j*| >= 5	377
Maximal violation	0.10 ± 0.01 Å
Torsion angle restraints (ϕ/ψ):	
Number	75
Maximal violation	2.94 ± 0.87°
Final CYANA target function value	2.61 ± 1.61 Å^2^
AMBER energy	−2208 ± 50 kcal/mol
RMSDs from ideal geometry:	
Bond lengths	0.015 ± 0.001 Å
Bond angles	1.93 ± 0.04°
RMSD to mean coordinates of residues 1–57:	
Backbone atoms N, C^α^, C'	0.37 ± 0.08Å
All heavy atoms	0.73 ± 0.08Å
PROCHECK Ramachandran plot statistics:	
Most favorable regions	78.5%
Additional allowed regions	21.5%
Generously allowed regions	0.0%
Disallowed regions	0.0%

**Figure 2:**
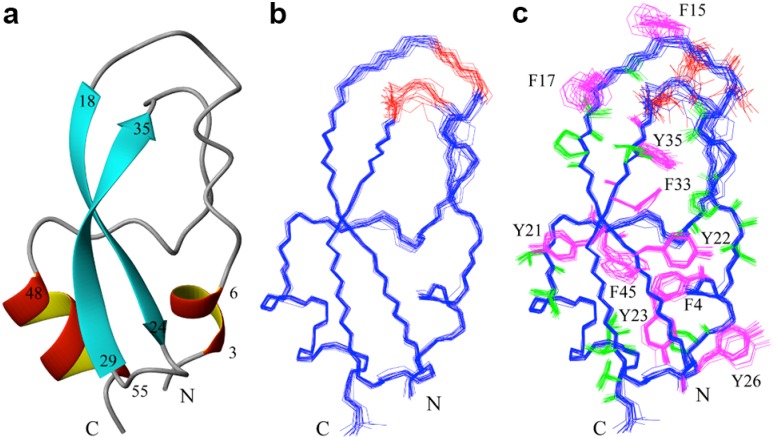
NMR solution structure of NACI. (**a**) Ribbon diagram of NACI. (**b**) Superposition of the backbone atoms of the 20 conformers that represent the solution structure of NACI for best fit of the backbone atoms N, C^α^, C’ in the secondary structure regions, residues 3–6, 18–24, 29–35, and 48–55. Residues Ser13–Cys14 and Ser36–Cys38 around the Cys14–Cys38 disulfide bond are not well defined due to missing resonances and indicated in red. (**c**) Side chains of Ser13–Cys14 and Ser36–Cys38 are shown in red, aromatic residues (F4, F15, F17, Y21, Y22, Y23, Y26, F33, Y35, F45) in magenta, and hydrophobic residues (Ala, Val, Leu, Ile, Thr, Pro) in green.

All (ϕ/ψ) backbone torsion angle pairs were found in the most favored or additionally allowed regions of the Ramachandran plot. NACI is an α/β protein with one a-helix (Ile48–Cys55) and two β-strands (Val18–Asn24 and Thr29–Tyr35) (Figure 2a). These two β-strands are arranged antiparallel as a β-sheet. In addition, Arg3–Glu6 form a 3_10_-helix. The ensembles fitted to the regions of secondary structure elements, residues 3–6, 18–24, 29–35 and 48–55, are shown in Figure 2b and c. The solution structure of NACI and the conformational restraints were deposited in the Protein Data Bank with accession code 2M99. The chemical shifts were deposited in the Biological Magnetic Resonance Data Bank (BMRB) [17] with accession number 19287.

**Figure 3:**
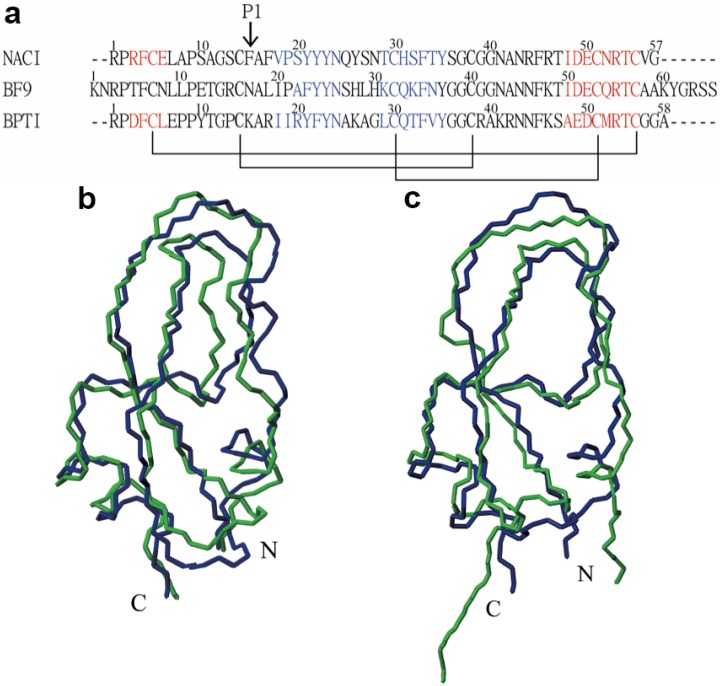
Sequence and structure comparison of NACI with homologues. (**a**) Sequence alignment of NACI with BF9 and BPTI. The secondary structure elements for NACI, BF9, and BPTI are shown in blue (β-strands) and red (helices). Disulfide bonds are shown with black lines. The arrow indicates the P1 residue. (**b**) Structural comparison between the mean structures of NACI (blue) and the BPTI (green). (**c**) Structural comparison between the mean structures of NACI (blue) and the BF9 (green).

In order to investigate the structural character of NACI, the secondary structures of NACI were compared with those in BPTI and BF9 based on sequence alignment by ClustalW2 (Figure 3a). NACI shows similar secondary structure elements as BPTI and BF9. With regard to the similarity of the three-dimensional fold between NACI and its homologues, comparisons of the solution structure of NACI with BPTI [18] and BF9 [5] showed that the RMSD values, calculated for the backbone atoms N, C^α^, and C’ in the secondary structure regions, between mean coordinates of NACI and its homologues are 1.09 Å for BPTI (PDB code: 1PIT) and 1.27 Å for BF9 (PDB code: 1JC6) (Figure 3b and c), indicating that NACI adopts a similar fold as BPTI and BF9. In particular, Figure 3b and c show that all three proteins share not only similar secondary structures but also the same tertiary fold.

**Figure 4:**
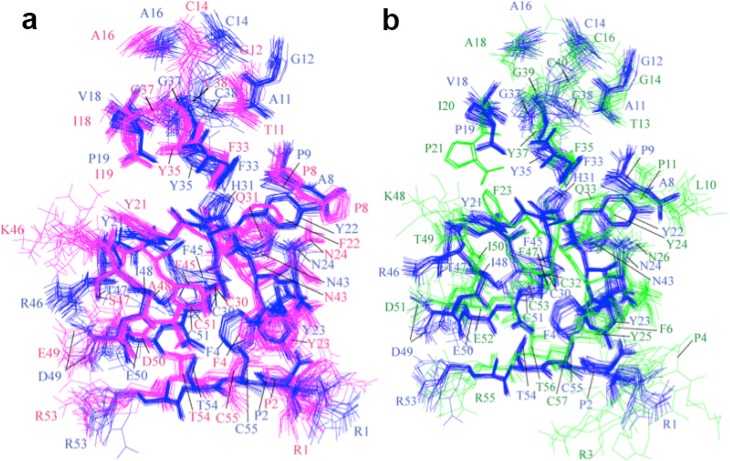
Comparison of residue-residue interactions between (**a**) NACI (blue) and BPTI (magenta) and (**b**) NACI (blue) and BF9 (green). Residues 1, 2, 4, 5, 8, 9, 11, 12, 14, 16, 18, 19, 21, 22, 23, 24, 30, 31, 33, 35, 37, 38, 43, 45, 46, 47, 48, 49, 50, 51, 53, 54, and 55 in NACI and BPTI as well as the corresponding residues in BF9 are shown.

Figure 4 compares residue-residue interactions between NACI and BPTI, and between NACI and BF9 for the conserved aromatic, hydrophobic, charged and polar amino acids. The side chain orientations of most conserved residues in NACI and BPTI as well as in NACI and BF9 coincide with each other, which suggests that the residue-residue interactions in NACI and BPTI as well as in NACI and BF9 are similar.

**Figure 5:**
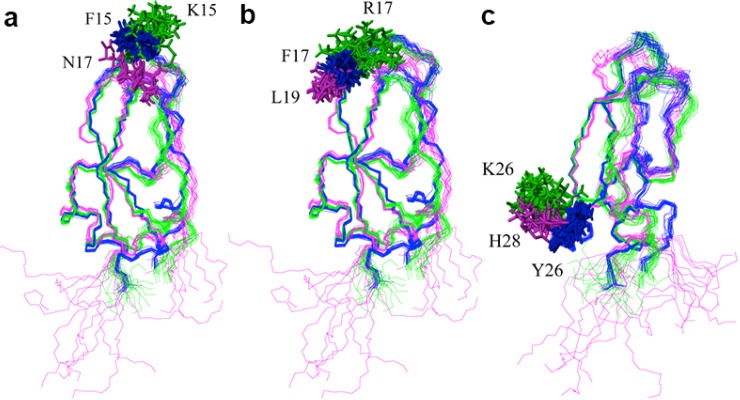
Side chain conformations of Phe15, Phe17, and Tyr26 in NACI (blue), and of corresponding residues in homologous structures. *i.e.*, Lys15, Arg17, and Lys26 in BPTI (green) and Asn17, Leu19, and His28 in BF9 (magenta). (**a**) Phe15 in NACI, Lys15 in BPTI, and Asn17 in BF9. (**b**) Phe17 in NACI, Arg17 in BPTI, and Leu19 in BF9. (**c**) Tyr26 in NACI, Lys26 in BPTI, and His28 in BF9.

NACI contains ten Tyr and Phe residues. Three of these side chains, Phe15, Phe17, and Phe45, are not converged to a defined conformation (Figure 2c). The disorder of the Phe45 side chain is due to missing ring resonances. The other two residues, Phe15, Phe17, as well as Tyr26, are not conserved based on the sequence alignment and point outside toward the solvent (Figure 2c), while the other seven Tyr and Phe residues are conserved as aromatic residues among NACI, BPTI, and BF9 (Figure 3a). Phe15, Phe17, and Tyr26 in NACI correspond to Asn17, Leu19, and His28 in BF9, and Lys15, Arg17, and Lys26 in BPTI. Comparisons of the side chain orientation of the three corresponding residues in BPTI, BF9 and NACI show that all these side chains in BF9 and BPTI point outside toward the solvent rather than into the interior of the proteins and do not show a well-defined conformation (Figure 5), which suggests that the three aromatic residues Phe15, Phe17, and Tyr26 are not essential for stabilizing the fold of NACI.

**Figure 6:**
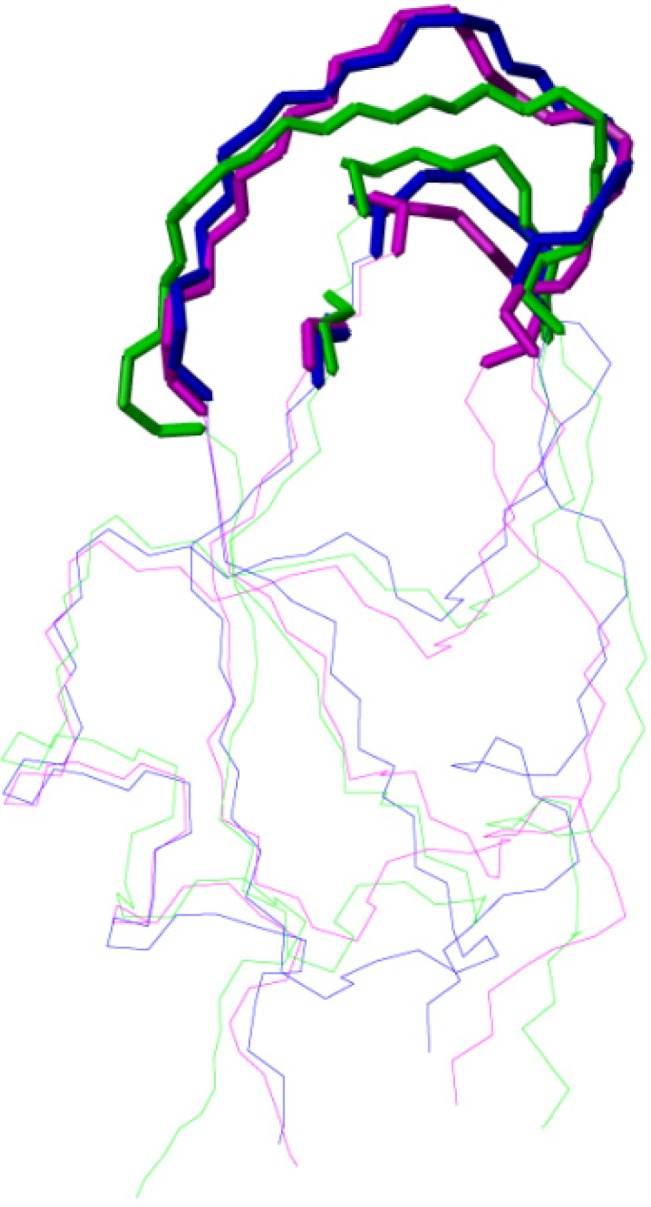
Backbone conformation of residues 11–19, 34, and 36–39 in the trypsin binding loop of BPTI (magenta), and corresponding residues in NACI (blue) and BF9 (green).

The functional site for the protease inhibitory activity is located in the binding loops. It was reported that BPTI binds to trypsin with residues 11–19, 34, and 36–39 [19]. Figure 6 shows that the backbone conformations of the binding loops in BPTI (magenta), NACI (blue), and BF9 (green) are similar, suggesting that the amino acid side chains in the binding loop regions might play an important role for the different inhibitory specificity of the three proteins.

**Figure 7:**
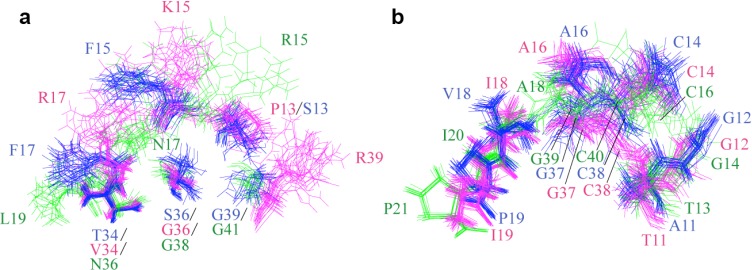
Side chains of residues 11–19, 34, and 36–39 in the trypsin binding loops of BPTI (magenta) and corresponding residues in NACI (blue) and BF9 (green). (**a**) Side chains of non-conserved residues. (**b**) Side chains of conserved residues.

Figure 7 shows the side chains in the binding loops, residues 11–19, 34, and 36–39. Ser13, Phe15, Phe17, Thr34, Ser36, and Gly39 in NACI (blue), corresponding to Pro13, Lys15, Arg17, Val34, Gly36, and Arg39 in BPTI (magenta), and Arg 15, Asn17, Leu19, Asn36, Gly38, and Gly41 in BF9 (green), are not conserved based on the sequence alignment (Figure 3a). Most side chains of these non-conserved residues, except Thr34 and Ser36, do not show a well-defined conformation (Figure 7a), which suggests that these residues might be involved in specificity, whereas Thr34 and Ser36 in NACI might participate in stabilizing a local conformation or contact with protease.

In BPTI an interior water molecule is located near the Cys14–Cys38 disulfide bond where it forms a hydrogen bond with the carbonyl-oxygen of Cys38, whereas in BPTI(G36S), conserved also as Ser36 in NACI, the hydroxyl side chain of Ser36 replaces the internal water molecule and forms hydrogen bonds with the same partners [9]. The non-conserved Ser36 in NACI might be involved in maintaining a certain conformation by a similar pattern as in the BPTI(G36S) mutant.

In contrast, the side chain conformations of the conserved residues are better converged (Figure 7b), except for residues 14, 37–38 in NACI and BPTI as well as 16, 39–40 in BF9, which is around Cys14–Cys38/Cys16–Cys40 disulfide bond region. The disorder of these conserved residues is probably due to isomerization of the Cys14–Cys38/Cys16–Cys40 disulfide bond.

One reported characteristic feature of the BPTI structure is the Cys14–Cys38 disulfide bond region, in which Cys14 is located in the primary binding loop and Cys38 in the second loop. It was reported that two conformational isomers due to isomerization of the Cys14–Cys38 disulfide bond were observed in BPTI and that the population of the minor form is 1.5% at 4 °C and increases to 8% at 68 °C [9]. In the NACI structure, regions around the Cys14–Cys38 disulfide bond are not well defined due to missing chemical shifts of residues Ser13–Cys14 and Ser36–Cys38 (Figure 2b and c). In addition, although H^α^ and H^β^ protons of Cys14 could be assigned, totally only two NOE restraints were assigned to Cys14, significantly less than the average of 20 NOE restraints per residue. The observed significant line broadening at 22 °C is confined to a small region around the Cys14–Cys38 disulfide bond, *i.e.*, residues Ser13–Cys14 and Ser36–Cys38, rather than being extended to longer segments in the primary binding loop and/or in the second loop, suggesting that the line broadening in NACI might result from conformational exchange around the Cys14–Cys38 disulfide bond. As reported in BPTI, isomerization of the Cys14–Cys38 disulfide bond might also exist in NACI. 

In an attempt to avoid the severe line broadening and to observe the signals from the major and minor conformers of the Ser13–Cys14 and Ser36–Cys38 residues, we measured HSQC spectra at different temperatures, 4, 10, 14, 22, 30, and 37 °C. It was reported that the population of the minor form in BPTI(G36S) is about 15%, *i.e.*, higher than in wild-type BPTI, and increases at higher temperatures [9]. NACI possesses also Ser36, as BPTI(G36S), suggesting that NACI might contain a higher population of the minor form than BPTI. Comparing HSQC spectra of NACI recorded at different temperatures showed that, in addition to some weak signals, few additional signals appeared, precluding assignments of a minor form.

## 3. Experimental

### 3.1. Sample Preparation

Recombinant NACI was obtained by overexpression in *Escherichia coli*. Protein expression and purification were described elsewhere [3]. The protein was uniformly labeled with ^15^N and ^13^C by growing bacteria on an isotope-enriched minimal medium using ^15^N ammonium chloride and ^13^C-enriched glucose as the principal nitrogen and carbon sources, respectively. NMR samples of purified protein (1–2 mM) were prepared in a buffer containing 50 mM sodium phosphate at pH 3.0 and 10% v/v ^2^H_2_O [5]. The pH value was chosen in agreement with that for the solution structure determination of the homologous chymotrypsin inhibitor BF9 [5], for which complete resonance assignments could not be obtained at neutral pH due to several missing backbone and side chain amide protons. In addition, BF9 was reported to possess similar conformations at different pH values [5]. The NACI protein sample was prepared by refolding before lowering the pH value to 3.0. Therefore the disulfide bonds had already formed when the pH was decreased to 3.0. The recombinant protein used for the NMR measurements comprised 89 amino acid residues. The C-terminal 57 residues corresponded to the Taiwan cobra chymotrypsin inhibitor, whereas the 32 non-native N-terminal residues were derived from the expression plasmid [3]. In principle, the N-terminal extension could be removed by thrombin cleavage. However, this was not done because it had been shown earlier [3] that only a marginal amount of NACI could be obtained after thrombin digestion and that the protein with the N-terminal extension was functional. The binding affinities of NACI to α-chymotrypsin were previously reported to be *K*_i_ = 180 and 133 nM with and without the N-terminal fused peptide, respectively [3]. 

### 3.2. NMR Measurements

The backbone assignment was based on 3D HNCACB, CBCA(CO)NH, HN(CA)CO, HCACO HBHA(CO)NH, and 2D ^15^N/^1^H-HSQC spectra. Side-chain ^1^H and ^13^C resonances were derived from 3D C(CO)NH and H(C)CH-TOCSY as well as heteronuclear 2D ^13^C/^1^H-CB(CGCD)HD and ^13^C/^1^H-CB(CGCDCE)HE. Homonuclear 2D NOESY and 3D ^15^N-resolved NOESY with 200 ms mixing time [5] were collected for protein structure determination. Spectra were recorded at 22 °C on a Varian VNMRS 600 MHz NMR spectrometer equipped with a ^1^H, ^15^N, ^13^C triple resonance probe head. The software packages VnmrJ and Sparky [20] were used for data processing and interactive spectra analysis, respectively.

### 3.3. Structure Calculation

Peak lists for the NOESY spectra were obtained by interactive peak picking with the program Sparky. The three-dimensional structure was determined by combined automated NOESY cross peak assignment [11] and structure calculation with torsion angle dynamics [13] implemented in the program CYANA [21]. Information of α-helices and β-strands were obtained from chemical shift index (CSI) analysis [22]. Restraints for hydrogen bonds involved in regular secondary structures were included. The hydrogen bonds between β-strands were included as restraints only if H^N^*_i_*-H^N^*_j_*, H^N^*_i_*-H^α^*_j_*_+1_, and H^α^*_i_*_−1_-H^α^*_j_*_+1_ NOE cross peaks between different β-strands were observed. The disulfide bonds used in the structure calculations were Cys5–Cys55, Cys14–Cys38, and Cys30–Cys51. Backbone torsion angle restraints were obtained with the program TALOS [23]. The standard CYANA protocol of seven iterative cycles of NOE assignment and structure calculation, followed by a final structure calculation, was applied. In each cycle the structure calculation started from 100 randomized conformers, and 8000 torsion angle dynamics steps were performed per conformer. The 20 conformers with the lowest final CYANA target function values were subjected to restrained energy refinement in explicit solvent against the AMBER force field [24] using the program OPALp [25,26].

## 4. Conclusions

The solution structure of the *Naja naja atra* chymotrypsin inhibitor (NACI) was determined by NMR. NACI shows similar secondary structure as two other serine protease inhibitors, the bovine pancreatic trypsin inhibitor (BPTI) and the *Bungarus fasciatus* fraction IX (BF9). Also their three-dimensional structures coincide closely with RMSD values of 1.09 Å between NACI and BPTI and 1.27 Å between NACI and BF9. In addition, broadening of resonances observed in NACI at 22 °C for residues Ser13–Cys14 and Ser36–Cys38, a region around the Cys14–Cys38 disulfide bond, suggests isomerization of the Cys14–Cys38 disulfide bond as reported for BPTI.
